# The relationship between neighborhood economic deprivation and asthma-associated emergency department visits in Maryland

**DOI:** 10.3389/falgy.2024.1381184

**Published:** 2024-06-05

**Authors:** Oluwasegun Akinyemi, Terhas Weldeslase, Eunice Odusanya, Mojisola Fasokun, Bukola Agboola, Tsion Andine, Esther Ayeni, Miriam Michael, Kakra Hughes

**Affiliations:** ^1^ Department of Surgery Outcomes Research Center, Howard University College of Medicine, Washington, DC, United States; ^2^ Department of Epidemiology, University of Alabama, Birmingham, AL, United States; ^3^ Maryland Institute for Applied Environmental Health, University of Maryland, College Park, MD, United States; ^4^ Department of Geography and Meteorology, Ball State University, Muncie, IN, United States; ^5^ Department of Internal Medicine, Howard University College of Medicine, Washington, DC, United States; ^6^ Department of Surgery, Howard University College of Medicine, Washington, DC, United States

**Keywords:** neighborhood economic deprivation, socioeconomic determinants of health, Distressed Communities Index (DCI), asthma, Emergency Department (ED) visits, state of Maryland, United States

## Abstract

**Background:**

Asthma represents a substantial public health challenge in the United States, affecting over 25 million adults. This study investigates the impact of neighborhood economic deprivation on asthma-associated Emergency Department (ED) visits in Maryland, using the Distressed Communities Index (DCI) for analysis.

**Methods:**

A retrospective analysis of Maryland's Emergency Department Databases from January 2018 to December 2020 was conducted, focusing on asthma-associated ED visits.

**Results:**

The study involved 185,317 ED visits, majority of which were females (56.3%) and non-Hispanic whites (65.2%). A significant association was found between increased neighborhood socioeconomic deprivation and asthma-related ED visits. The poorest neighborhoods showed the highest rates of such visits. Compared to prosperous areas, neighborhoods classified from Comfortable to Distressed had progressively higher odds for asthma-related ED visits (Comfortable: OR = 1.14, Distressed OR = 1.65). Other significant asthma predictors included obesity, female gender, tobacco smoking, and older age.

**Conclusion:**

There is a substantive association between higher asthma-related ED visits and high neighborhood economic deprivation, underscoring the impact of socioeconomic factors on health outcomes.

**Public health implications:**

Addressing healthcare disparities and improving access to care in economically distressed neighborhoods is crucial. Targeted interventions, such as community health clinics and asthma education programs, can help mitigate the impact of neighborhood disadvantage.

## Introduction

Asthma represents a substantial public health challenge in the United States, affecting over 25 million adults (1). This chronic respiratory condition, characterized by persistent inflammation and airway constriction, imposes a considerable burden on individuals and the healthcare system. The link between socioeconomic factors, environmental influences, and the development of chronic illnesses, such as asthma, is increasingly recognized as a critical determinant of health outcomes (2). The present study delves into the multifaceted burden of asthma in the United States, encompassing prevalence, healthcare costs, and the intricate interplay of socioeconomic disparities, pollution, climate change, and neighborhood poverty (3, 4).

The pathophysiology of asthma involves complex cellular mechanisms, particularly influenced by the Th2-type immune response, which plays a central role in mediating inflammatory processes in the airways (5). The transcription factor GATA-3 is pivotal in these mechanisms, orchestrating the differentiation and function of Th2 cells that secrete key cytokines such as interleukin-13 (IL-13), IL-4, IL-5, and IL-9 (6–8). These cytokines contribute to characteristic asthma symptoms, including airway hyperresponsiveness, inflammation, and mucus production. IL-13, especially, has been identified as a critical mediator, influencing various cellular activities that lead to asthma's clinical manifestations (9, 10). It promotes the hypersecretion of mucus, airway fibrosis, and an increase in eosinophilic inflammation within the airway tissues. Targeting IL-13 and GATA-3 provides a potential therapeutic approach, given their significant roles in the exacerbation of asthma symptoms (6, 11).

Asthma's etiology is further complex and multifactorial, with environmental factors playing a pivotal role. Environmental pollution, climate change, and degradation of natural ecosystems are closely linked to the increased prevalence of asthma in recent decades (12). Pollutants such as particulate matter, ozone, and allergens exacerbate respiratory conditions and can trigger asthma attacks (11). Furthermore, climate change contributes to the proliferation of allergenic plants and intensifies extreme weather events, potentially increasing the burden of asthma (12). Importantly, these environmental factors are often intertwined with neighborhood poverty.

Asthma is not merely a health concern; it is a substantial economic burden in the United States. The prevalence of asthma has been steadily increasing and it is linked to substantial healthcare expenditures, accounting for both direct and indirect costs (13–15). Direct costs encompass medical expenses associated with doctor's visits, hospitalizations, and medications, while indirect costs entail productivity losses due to missed workdays, decreased job performance, and the strain on caregivers. American taxpayers bear a significant portion of this financial burden, with asthma-related healthcare costs estimated to exceed $80 billion annually (16). Additionally, absenteeism from work and reduced productivity due to asthma result in substantial losses for both individuals and employers (17).

The socio-economic context in which individuals reside significantly impacts their health outcomes. Neighborhoods characterized by economic deprivation often face greater exposure to environmental hazards (18), inadequate access to healthcare services, and limited resources for health promotion and disease management (19). The study highlights the critical interplay between neighborhood economic deprivation, pollution, and asthma-related emergency department (ED) visits. Investigating these relationships in the context of Maryland provides valuable insights into regional variations and underscores the urgency of addressing health disparities.

The study aims to explore the intricate relationship between neighborhood socioeconomic deprivation, as measured by the Distressed Communities Index (DCI), and asthma-associated ED visits in Maryland. In this retrospective analysis of a statewide data, we seek to understand how economic disparities within neighborhoods influence asthma-related hospital visits. Additionally, we explore common risk factors that contribute to the development of asthma, considering the broader context of healthcare disparities and environmental influences.

## Materials and methods

### Study population

As of 2022, Maryland hosts approximately 6.2 million residents, characterized by a rich tapestry of ethnic and economic diversity. The ethnic composition includes White (57.3%), Black (31.7%), Asian (7.1%), and Hispanic (11.1%) populations. This diversity extends into the workforce, represented across a range of industries including construction, educational services, and food services, with substantial contributions from higher-paying sectors such as public administration and finance. Despite a relatively high median household income of $91,431 in 2021, Maryland experiences notable income disparities. The Gini index of 0.456, although lower than the national average, underscores ongoing income inequality. Additionally, gender disparities in earnings persist across different sectors (20).

### Study dataset

This study utilized the Maryland State Emergency Department Databases (SEDD) from the Healthcare Cost and Utilization Project (HCUP). The SEDD documents discharge information for all emergency department (ED) visits not leading to an admission, while cases that result in hospital stays are recorded in the State Inpatient Databases (SID). These data provide statewide estimates of ED visits, useful for comparisons and analyzing rare conditions due to its extensive sample size. The comprehensive dataset also enables robust analysis of ED utilization patterns (21).

### Study design

The study employed a retrospective analysis to examine the association between community-level socioeconomic deprivation and ED visits for Asthma in Maryland from January 2018 to December 2020. Using data from the Maryland SEDD (21), we utilized the DCI to measure socioeconomic deprivation at the community level. Multivariate logistic regression analyses were conducted to explore the association between the DCI categories—prosperous, comfortable, mid-tier, at-risk, and distressed—and the likelihood of ED visits due to Asthma. The study objective was to clarify how neighborhood socioeconomic factors influence ED visits due to Asthma.

### Inclusion and exclusion criteria

Inclusion criteria were defined as all patients residing in Maryland who visited the emergency department with a primary diagnosis of acute asthma between January 2018 and December 2020. Exclusion criteria included individuals younger than 18 years or older than 85 years, as well as patients lacking zip code information.

### Outcome variable

The primary outcome variable of interest in this study was the occurrence of asthma-related ED visits. These ED visits were identified based on diagnostic codes and records indicating asthma-related complaints and treatment (see Supplementary File).

### Variable of interest

The variable of primary interest in this analysis was the Distressed Communities Index (DCI). The DCI is a diagnostic tool that classifies American communities into five levels of economic vitality: prosperous, comfortable, mid-tier, at-risk, and distressed. Developed using the U.S. Census Bureau's American Community Survey and Business Patterns data, the DCI maps the economic status of zip codes, counties, and congressional districts to reveal disparities in economic well-being across and within states. This index evaluates communities through seven critical metrics: the proportion of adults over 25 without a high school diploma, the housing vacancy rate, the percentage of the prime-age population that is not employed, the poverty rate, the median household income relative to the broader metro or state area, and the changes over the past five years in employment and business establishments (22).

These metrics are averaged and equally weighted to calculate each area's preliminary score, which is then normalized to create a final Distress Score ranging from 0 to 100, where 0 represents the most prosperous communities and 100 denotes the most distressed. The index covers 99% of the U.S. population, involving about 26,000 zip codes with at least 500 residents, although it excludes U.S. territories such as Puerto Rico due to data limitations. The DCI employs ZIP Code Tabulation Areas (ZCTAs), approximations of postal zip codes that facilitate economic and demographic analysis at the sub-county level, despite the potential mismatches in geographical boundaries that can change over time. This meticulous approach allows the DCI to provide a nuanced view of the socio-economic landscapes across various American communities, aiding policymakers, researchers, and the public in identifying areas in need of attention and resources (22).

### Covariates

In our analysis, we integrated several key covariates to address potential confounders and better understand their impacts on asthma-related ED visits. Demographic characteristics such as age (categorized as under 45, 45–65, and over 65 years), gender, and race/ethnicity were considered due to their known influences on health outcomes. We also evaluated the role of insurance status (private, Medicare, Medicaid, self-pay, and others) and timing of ED visits (weekends vs. weekdays). Health conditions, including common comorbidities like hypertension, diabetes, HIV, anxiety, depression, obesity, and substance use (tobacco and alcohol), were factored into the analysis. These covariates were essential for the final multivariable logistic regression, aiming to highlight the independent association between neighborhood socioeconomic poverty captured by the DCI and asthma-related ED visits.

### Geospatial analysis and data integration for environmental health assessment

We analyzed data using ArcGIS software from two key sources: the CDC Environmental Justice Index and the EPA Environmental Justice Screening and Mapping Tool. The CDC Environmental Justice Index (EJI) is a national tool designed to quantify the overall effects of environmental burden while considering human health and health equity (23). Similarly, the EPA EJScreen is a national tool designed by the Environmental Protection Agency to identify regions that might require further resource allocation (24). We downloaded the CSV File of the national environmental justice Screen data at the tract level, and the EPA's Environmental Justice mapping tool Geodatabase, at the tract level. Geodatabase is the main data format for editing and managing data in ArcGIS, the software's original data structure (24). The data was added into the ArcGIS software, and the maps were symbolized using graduated colors, and desired fields (25). The maps used in this study are also referred to as choropleth maps.

### Statistical analysis

Categorical variables were presented as frequencies and percentages. Pearson's Chi-square test was used to stratify study variables across asthma-related and non-asthma-related ED visits. We included variables that showed statistically significant associations in the multivariate analysis.

A logistic regression analysis was employed to assess the association between the DCI and asthma-related ED visits while controlling for covariates. Odds ratios (ORs) with corresponding 95% confidence intervals (CIs) were calculated to quantify the strength and direction of these associations. Statistical significance was determined at a *P*-value threshold of 0.05. This comprehensive statistical approach allowed us to explore the multifaceted relationship between neighborhood socioeconomic status, individual-level factors, and ED visits for asthma within the Maryland population during the specified timeframe.

## Results

The bivariate analysis of our large cohort (*n* = 1,665,516), of whom 185,317 (11.1%) experienced asthma-related ED visits, revealed significant associations between various demographic, socioeconomic, and health-related factors and asthma-related visits. Age showed a strong association with asthma-related ED visits, as individuals over 65 years had the highest prevalence (61.2%), while those aged 18–45 years had the lowest (6.2%) (*P* < 0.001). Females comprised 56.3% of the asthma group compared to 56.8% in the non-asthma group (*P* < 0.001) (Table 1).

**Table 1:** Baseline characteristics of study population.

	All ED visits	Asthma-related ED visits	Non-asthma ED visits	*P* value
	(*n* = 1,665,516)	(*n* = 185,317)	(*n* = 1,480,199)	
Age (Years)				<0.001
18–45	636,623 (38.2%)	11,517 (6.2%)	625,106 (42.2%)	
45–65	416,082 (25.0%)	60,400 (32.6%)	355,682 (24.0%)	
>65	612,811 (36.8%)	113,400 (61.2%)	499,411 (33.7%)	
Female	945,257 (56.8%)	104,385 (56.3%)	840,872 (56.8%)	<0.001
Race/ethnicity				<0.001
White	879,693 (53.5%)	119,981 (65.2%)	759,712 (52.0%)	
Black	551,068 (33.5%)	57,487 (31.2%)	493,581 (33.8%)	
Hispanics	120,233 (7.3%)	2,681 (1.5%)	117,552 (8.0%)	
Asian/Pacific Islander	48,953 (3.0%)	1,610 (0.9%)	47,343 (3.2%)	
Native Americans	3,133 (0.2%)	292 (0.2%)	2,841 (0.2%)	
Others	42,671 (2.6%)	1,985 (1.1%)	40,686 (2.8%)	
Insurance				<0.001
Medicare	671,615 (41.6%)	126,503 (69.8%)	545,112 (38.0%)	
Medicaid	409,426 (25.3%)	29,334 (16.2%)	380,092 (26.5%)	
Private	505,571 (31.3%)	24,201 (13.4%)	481,370 (33.6%)	
Uninsured	25,117 (1.6%)	995 (0.6%)	24,122 (1.7%)	
Others	3,924 (0.2%)	126 (0.1%)	3,798 (0.3%)	
Income				<0.001
Quartile I	206,598 (12.3%)	7,991 (13.1%)	198,607 (12.3%)	
Quartile II	221,673 (13.2%)	7,804 (12.8%)	213,869 (13.2%)	
Quartile III	524,801 (31.2%)	19,499 (32.0%)	505,302 (31.2%)	
Quartile IV	728,257 (43.3%)	25,671 (42.1%)	702,586 (43.4%)	
Distressed community index				<0.001
Prosperous	384,635 (25.5%)	34,489 (20.5%)	350,146 (26.1%)	
Comfortable	391,642 (25.9%)	41,405 (24.6%)	350,237 (26.1%)	
Mid-tier	342,404 (22.7%)	35,358 (21.0%)	307,046 (22.9%)	
At risk	173,489 (11.5%)	22,565 (13.4%)	150,924 (11.2%)	
Distressed	218,942 (14.5%)	34,496 (20.5%)	184,446 (13.7%)	
Comorbidities				
Current smokers	312,036 (18.7%)	69,856 (37.7%)	242,180 (16.4%)	<0.001
Hypertension	517,391 (31.1%)	72,747 (39.3%)	444,644 (30.4%)	<0.001
Diabetes Mellitus	289,241 (17.4%)	46,908 (25.3%)	242,333 (16.4%)	<0.001
HIV	20,344 (1.2%)	3,488 (1.9%)	16,856 (1.1%)	<0.001
Obesity	376,214 (22.6%)	171,070 (92.3%)	205,144 (13.9%)	<0.001
Other conditions				
Alcohol addiction	67,269 (4.0%)	9,031 (4.9%)	58,238 (3.9%)	<0.001
Depression	236,561 (14.2%)	38,563 (20.8%)	197,998 (13.4%)	<0.001
Weekend	354,480 (21.3%)	42,234 (22.8%)	312,246 (21.1%)	<0.001
Anxiety	243,600 (14.6%)	41,464 (22.4%)	202,136 (13.7%)	<0.001
Pneumonia	59,509 (3.6%)	13,437 (7.3%)	46,072 (3.1%)	<0.001

Racial and ethnic differences were evident, with white individuals constituting a higher proportion of the asthma group (65.2%) compared to the non-asthma group (52.0%). Conversely, Hispanics and Asian/Pacific Islanders had a lower representation in the asthma group compared to their non-asthma counterparts (*P* < 0.001).

Insurance status was significantly associated with asthma-related ED visits, with a higher proportion of individuals with asthma covered by Medicare (69.8% vs. 38.0% in non-asthma) and a lower proportion having private insurance (13.4% vs. 33.6% in non-asthma) (*P* < 0.001).

The DCI revealed that individuals from “prosperous” communities had a lower prevalence of asthma compared to those from “distressed” communities (*P* < 0.001). Regarding comorbidities, there was a higher prevalence of current smokers, hypertension, diabetes mellitus, HIV, and obesity in the asthma group compared to the non-asthma group (*P* < 0.001 for all). Additionally, asthma was more prevalent in individuals with alcohol addiction, depression, anxiety, and pneumonia (*P* < 0.001 for each).

**Figure 1:**
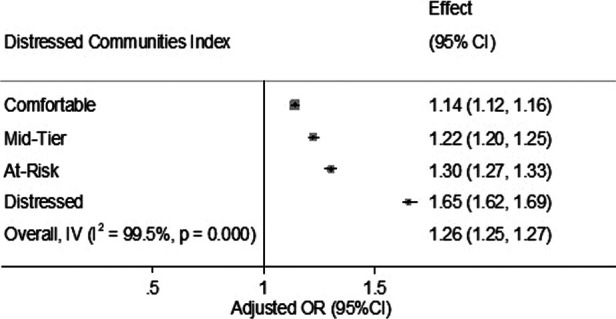
Association between the distressed communities Index and asthma related ED visits in Maryland (2018–2020): the forest plot illustrates the relationship between community distress levels and the likelihood of a specific outcome, expressed as adjusted odds ratios (OR) with 95% confidence intervals (CI). The analysis shows increasing odds of the outcome as community distress levels rise, with “Distressed” communities showing the highest odds ratio at 1.65 (95% CI 1.62–1.69). Overall effect across all categories is an OR of 1.26 (95% CI 1.25–1.27), with a high degree of heterogeneity (*I*^2^ = 99.5%, *P* = 0.000), indicating substantial variability in effects across studied groups.

Our regression analysis (Table 2) examined the factors associated with asthma-related ED visits, revealing significant associations for various covariates:

Age—Compared to adults aged 18–45 years (reference), individuals aged 45–64 years had a substantially higher odds ratio (OR) of 3.36 (95% CI 3.28–3.45), and those aged over 65 years had an even higher OR of 3.84 (95% CI 3.73–3.96) for ED visits related to asthma.
Gender—Females had a slightly increased risk of ED visits for asthma with an OR of 1.13 (95% CI 1.11–1.14).
Health Conditions—Several health conditions were associated with increased odds of ED visits for asthma, including HIV (OR = 1.57, 95% CI 1.49–1.66), anxiety (OR = 1.23, 95% CI 1.21–1.25), depression (OR = 1.05, 95% CI 1.03–1.06), and tobacco use (OR = 1.40, 95% CI 1.38–1.42). Additionally, a history of pneumonia was associated with an increased risk (OR = 1.45, 95% CI 1.41–1.49) of ED visits for asthma.
Insurance—Individuals covered by Medicare (OR = 1.90, 95% CI 1.85–1.94), Medicaid (OR = 2.04, 95% CI 2.00–2.09), or other types of insurance (OR = 1.65, 95% CI 1.32–2.06) had higher odds of ED visits for asthma compared to those with private insurance (reference). Self-pay patients also had an increased risk of asthma-related ED visits (OR = 1.17, 95% CI 1.07–1.27).
Neighborhood Socioeconomic Status (Figure 1)—Neighborhoods classified as Mid-Tier (OR = 1.22, 95% CI 1.20–1.25), At-Risk (OR = 1.30, 95% CI 1.27–1.33), and Distressed (OR = 1.65, 95% CI 1.62–1.69) had significantly higher odds of ED visits for asthma compared to Prosperous neighborhoods (reference). Comfortable neighborhoods also exhibited a modestly increased risk (OR = 1.14, 95% CI 1.12–1.16).
Race/Ethnicity—Ethnicity played a role, with Black individuals having lower odds (OR = 0.82, 95% CI 0.81–0.84) of ED visits for asthma compared to White individuals (reference). Hispanic individuals had substantially lower odds (OR = 0.41, 95% CI 0.39–0.43), while Pacific Islanders (OR = 0.80, 95% CI 0.76–0.86) and individuals of other ethnicities (OR = 0.72, 95% CI 0.68–0.76) also had lower odds. Native Americans did not show a significant difference in odds (OR = 0.92, 95% CI 0.78–1.09).
Weekend Admissions—ED visits for asthma on weekends had slightly higher odds (OR = 1.12, 95% CI 1.11–1.14) compared to weekday admissions.
Substance Abuse—Substance abuse did not significantly affect the odds of ED visits for asthma (OR = 0.81, 95% CI 0.34–1.94).
Alcohol Use—Alcohol use was associated with higher odds of ED visits for asthma (OR = 1.34, 95% CI 1.30–1.39).
Obesity—Obesity was strongly associated with increased odds of ED visits for asthma, with an exceptionally high OR of 53.13 (95% CI 52.13–54.16).

**Table 2:** Risk factors for asthma-related ED visits in Maryland.

Asthma	Odds Ratio	Lower CI	Upper CI	*P*-value
Age				
18–45 Years	Reference			
45–64 years	3.36	3.28	3.45	<0.001
>65 Years	3.84	3.73	3.96	<0.001
Female	1.13	1.11	1.14	<0.001
HIV	1.57	1.49	1.66	<0.001
Anxiety	1.23	1.21	1.25	<0.001
Depression	1.05	1.03	1.06	<0.001
Tobacco	1.40	1.38	1.42	<0.001
Pneumonia	1.45	1.41	1.49	<0.001
Insurance				
Private	Reference			
Medicare	1.90	1.85	1.94	<0.001
Medicaid	2.04	2.00	2.09	<0.001
Uninsured	1.17	1.07	1.27	<0.001
Others	1.65	1.32	2.06	<0.001
Distressed community index				
Prosperous	Reference			
Comfortable	1.14	1.12	1.16	<0.001
Mid-Tier	1.22	1.20	1.25	<0.001
At-Risk	1.30	1.27	1.33	<0.001
Distressed	1.65	1.62	1.69	<0.001
Race/ethnicity				
White	Reference			
Black	0.82	0.81	0.84	<0.001
Hispanic	0.41	0.39	0.43	<0.001
Pacific Islanders	0.80	0.76	0.86	<0.001
Native Americans	0.92	0.78	1.09	0.33
Others	0.72	0.68	0.76	<0.001
Weekend Admissions	1.12	1.11	1.14	<0.001
Substance Abuse	0.81	0.34	1.94	0.64
Alcohol	1.34	1.30	1.39	<0.001
Obesity	53.13	52.13	54.16	<0.001
_cons	0.00	0.00	0.00	<0.001

**Figure 2:**
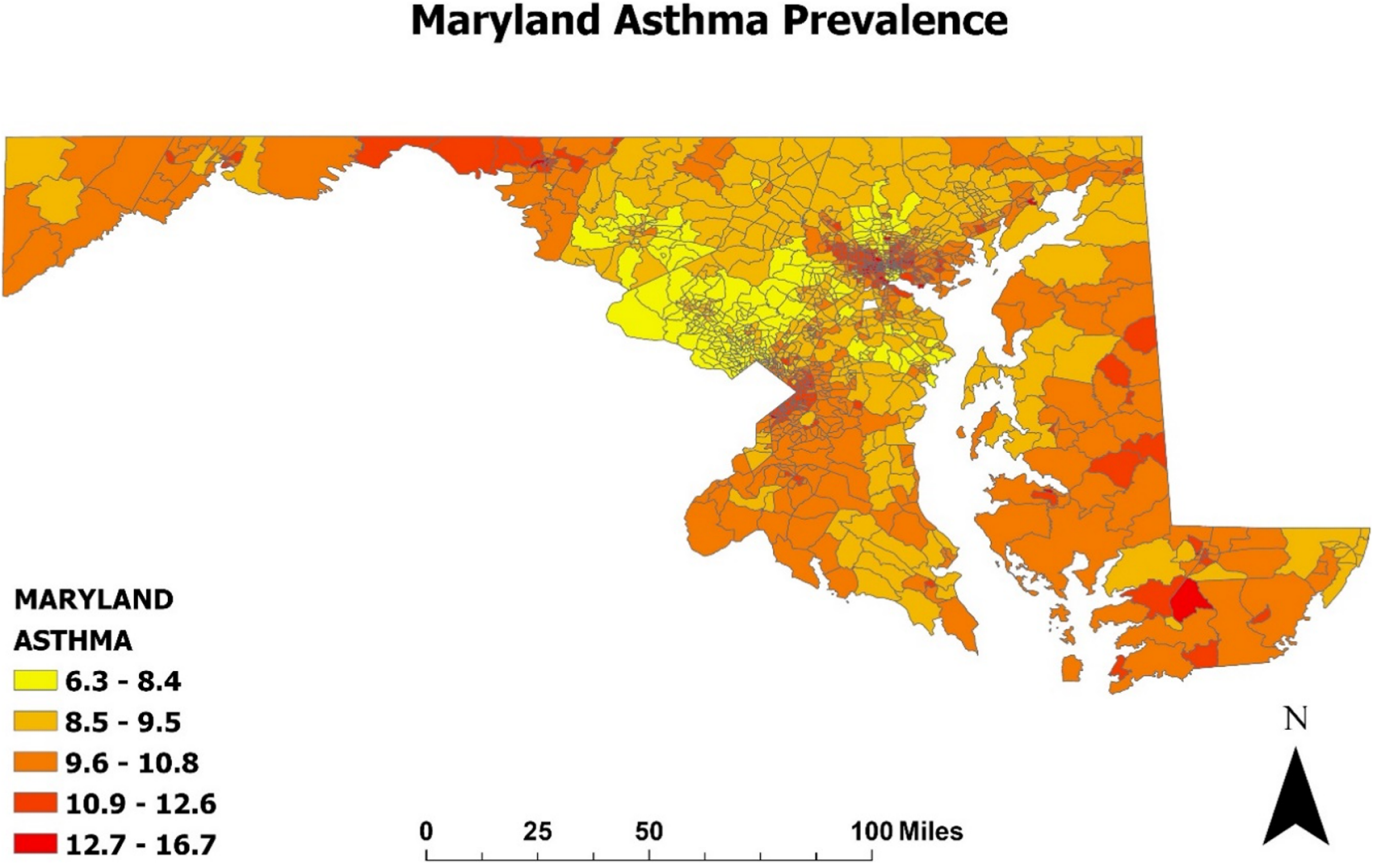
Prevalence of asthma in Maryland (2020): Map of the incidence of asthma in Maryland was developed using ArcGIS software and data extracted from the CDC environmental justice Index. The map is displayed at the census tract level, which are subdivisions of counties used by the Census to gather statistical data. Higher levels of exposure are indicated by darker colors on the map and the legend illustrates the percentage of Maryland residents with asthma.

**Figure 3:**
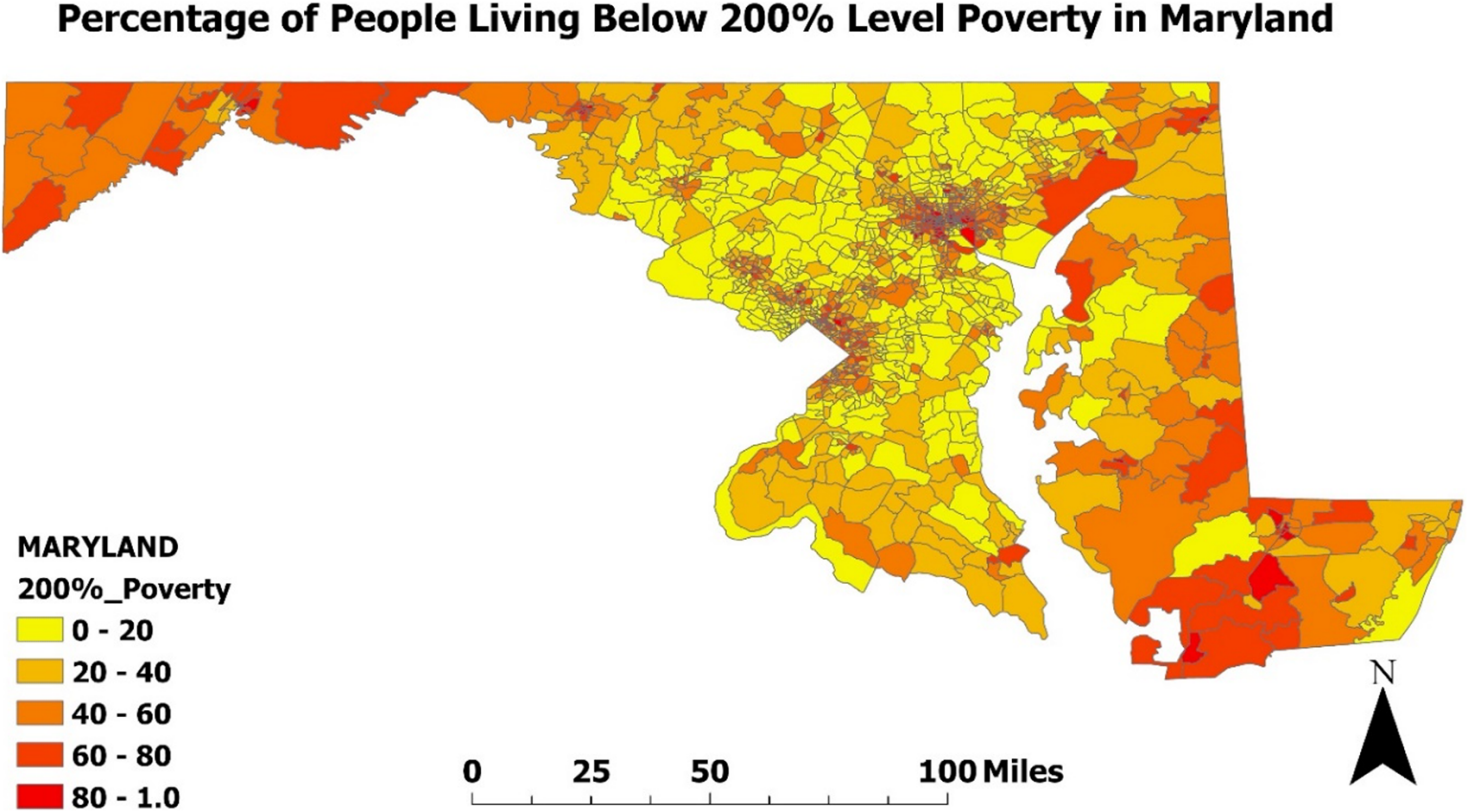
Percentage of people living below poverty level in Maryland (2020): Map of the percentage of people living below the 200% poverty level in Maryland was developed using ArcGIS software and data extracted from the CDC environmental justice Index. The map is displayed at the census tract level, which are subdivisions of counties used by the Census to gather statistical data. Darker colors on the map represent a higher percentage of poverty.

**Figure 4:**
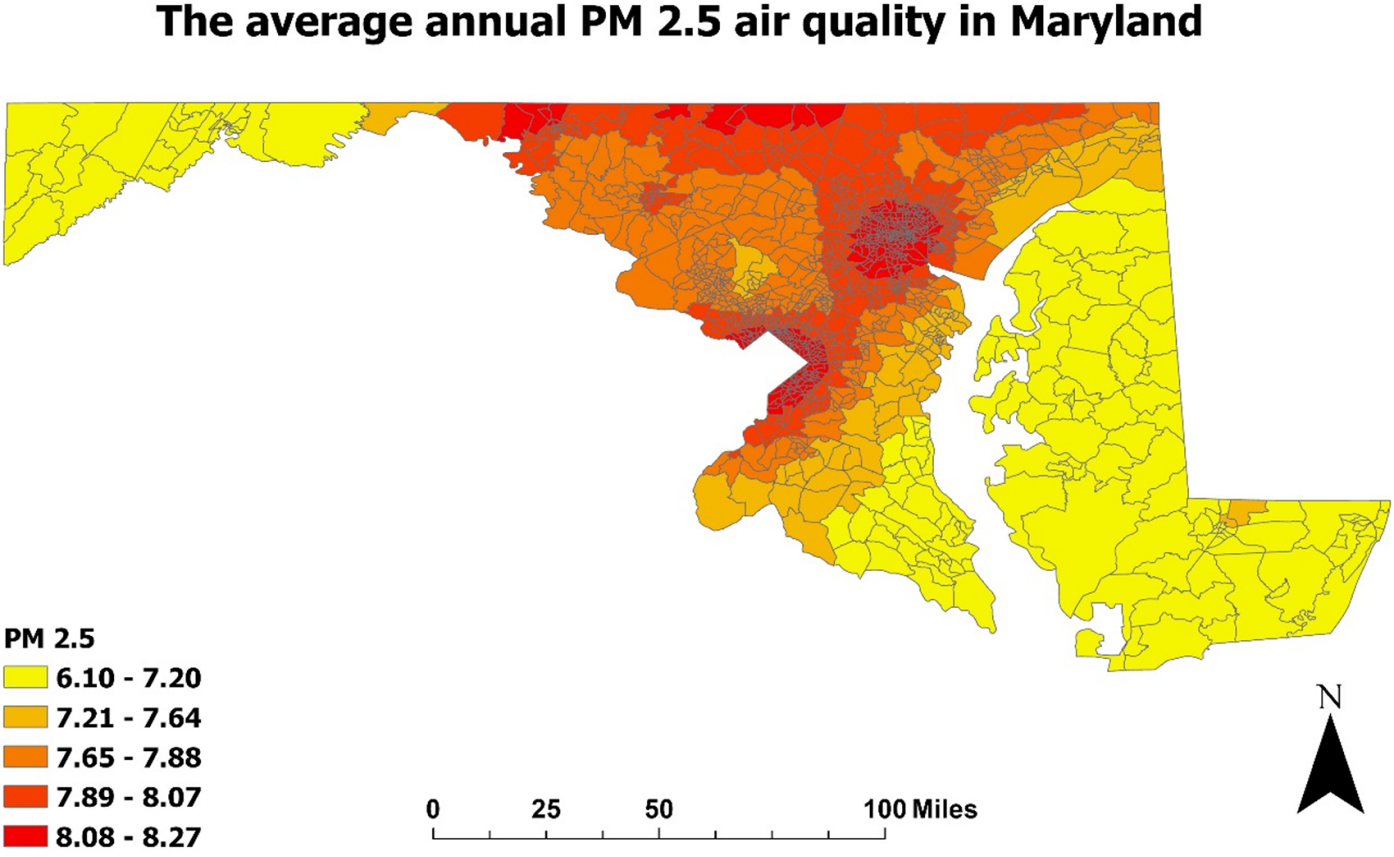
Air pollution level in Maryland (2020): Map of the annual average of PM 2.5 level in the air in Maryland was developed using ArcGIS software and data extracted from the EPA environmental justice and screening mapping tool (EPA EJScreen). PM 2.5 is defined by the particulate matter combination of solid and liquid droplets in the air with a diameter of 2.5 micrometers or less.

In a sub analysis shown in Figures 2–4, we examined the prevalence of asthma in Maryland in 2020 in relation to poverty and air pollution levels (23, 24, 29, 30). Figures 2, 3 highlight a striking correlation between asthma prevalence and poverty, with the highest incidence of asthma found in the poorest neighborhoods. This is based on data from the CDC indicating the percentage of people living below 200% on the poverty line. Figure 4, focusing on air pollution using PM 2.5 Particulate levels, reveals a less pronounced but still notable association between air pollution and asthma prevalence. Interestingly, areas with the highest air pollution did not consistently report the highest asthma prevalence. These findings, corroborating our hospital data, suggest that neighborhood poverty may be a more significant predictor of asthma-related ED visits than air pollution alone.

## Discussion

The present study revealed a significant association between neighborhood socioeconomic status, as measured by the Distressed Communities Index, and ED visits due to asthma. Specifically, as neighborhood economic deprivation increased, there was a corresponding increase in the likelihood of ED visits for asthma. The odds of such visits were notably higher in neighborhoods classified as At-Risk or Distressed compared to Prosperous areas.

Our findings align with a growing body of literature that underscores the critical role of neighborhood socioeconomic status in shaping health outcomes (30–32). Residents of economically disadvantaged neighborhoods often face numerous challenges, including limited access to healthcare resources, exposure to environmental pollutants, and increased psychosocial stressors. These factors can collectively contribute to a higher prevalence of asthma and a greater likelihood of ED visits.

Environmental Pollution and Neighborhood Poverty—Environmental pollution, often exacerbated in economically distressed neighborhoods, has been strongly linked to the development and exacerbation of asthma. Pollutants such as particulate matter, ozone, and allergens are known triggers for asthma attacks. Residents in impoverished neighborhoods are more likely to reside near industrial facilities, highways, or areas with suboptimal air quality, increasing their exposure to these harmful pollutants (18). Our study underscores the interconnectedness of environmental factors and neighborhood poverty in asthma-related ED visits.

Our study reveals a substantive association between asthma-related ED visits and neighborhood poverty in Maryland than between asthma and air pollution. This aligns with literature underscoring socio-economic influences on health (33) but diverges from some research emphasizing air pollution as a primary asthma determinant. The heightened asthma incidence in poorer neighborhoods likely results from multifaceted factors like inadequate healthcare access, substandard living conditions, and increased stress, all known asthma triggers. This finding suggests compounded environmental and socio-economic risks in these areas.

The less pronounced association between asthma and air pollution, compared to neighborhood poverty, highlights our study's nuanced approach, considering socio-economic variables alongside environmental factors. This contrasts with some studies focusing primarily on environmental determinants (34).

The association between neighborhood disadvantage and asthma-related ED visits can be attributed to various factors:

Access to Healthcare—Residents in economically distressed neighborhoods may have limited access to quality healthcare services (35), including preventive care and asthma management. Delayed or inadequate treatment can lead to the exacerbation of asthma symptoms, prompting ED visits.
Environmental Exposures—As mentioned, polluted environments in economically disadvantaged neighborhoods can worsen asthma symptoms and increase the need for emergency care (34).
Psychosocial Stressors—High levels of neighborhood poverty are often accompanied by psychosocial stressors, which can contribute to the development and worsening of asthma (36, 37). Chronic stress may lead to immune system dysregulation and exacerbate asthma symptoms (26).
Health Behavior and Lifestyle—Residents in disadvantaged neighborhoods may have limited resources for maintaining a healthy lifestyle, including proper nutrition and exercise. Unhealthy behaviors, such as tobacco smoking and poor diet, can increase the risk of asthma (27).

### Implications of findings

Our findings have several implications for public health and healthcare policy:

Targeted Interventions—Addressing healthcare disparities and improving access to care in economically distressed neighborhoods is crucial. Targeted interventions, such as community health clinics and asthma education programs, can help mitigate the impact of neighborhood disadvantage.

These findings suggest that addressing socioeconomic disparities could be more impactful for asthma prevention and management than solely focusing on environmental interventions. Our exploration of both socio-economic and environmental factors offers a more holistic view of asthma-related ED visits, potentially guiding more effective public health strategies. By focusing on broader socio-economic issues, health policies can more effectively target the root causes of asthma in impoverished communities.

Environmental Policies—Policies aimed at reducing environmental pollution and promoting clean air initiatives are essential for reducing asthma-related ED visits. Investments in cleaner energy sources and transportation alternatives can benefit vulnerable communities.
Health Education—Raising awareness about asthma management, including the importance of regular asthma check-ups and medication adherence, can empower individuals in economically disadvantaged neighborhoods to better manage their condition and potentially reduce ED visits.

### Association with other factors

In this study, we observed that older age groups, specifically those aged 45–64 years and those over 65 years, had significantly higher odds of ED visits for asthma compared to younger adults aged 18–44 years. This trend aligns with the documented higher prevalence of asthma in older populations, as noted in previous studies (27). Additionally, gender differences were evident, with females showing slightly higher odds of ED visits for asthma than males. This observation is consistent with prior research indicating a greater prevalence of asthma among women (23, 28).

Insurance status also played a crucial role in the likelihood of asthma visits to the ED. Individuals covered by Medicaid and Medicare had significantly higher odds of such visits than those with private insurance, underscoring the critical role of insurance coverage in the management of chronic conditions like asthma.

Regarding racial and ethnic disparities, our findings revealed that Non-Hispanic Blacks and Hispanics were less likely to visit the ED for asthma compared to Non-Hispanic whites. The contradictory findings of higher asthma-related ED visits among residents of the poorest neighborhoods and Medicaid beneficiaries, yet also higher among whites, could be due to various factors. It is possible that while poverty and Medicaid status are strong indicators of asthma-related ED visits, there may be additional factors at play among white individuals, such as differential access to healthcare, healthcare-seeking behaviors, or environmental exposures, that contribute to higher rates of ED visits in this group despite their socioeconomic status.

We also noted that admissions during weekends were associated with slightly higher odds of ED visits for asthma, suggesting potential variations in healthcare access and management on weekends.

Finally, the strong association between obesity and asthma-related ED visits emphasizes the need to address comorbid conditions and lifestyle factors in asthma management. This association highlights the complex interplay between chronic disease management and broader health determinants.

Together, these findings highlight several factors that influence the frequency of ED visits for asthma and underscore the need for targeted interventions that consider age, gender, insurance coverage, racial and ethnic backgrounds, timing of hospital admissions, and associated health conditions such as obesity.

### Limitations

This study relies on administrative data from the Maryland Emergency Department Sample Database, which may introduce limitations related to data accuracy and completeness. The data primarily serve billing and administrative purposes, potentially leading to underreporting and misclassification of asthma-related ED visits. Additionally, our findings may have limited generalizability beyond Maryland, as regional healthcare infrastructure and socioeconomic conditions can vary significantly. The study's cross-sectional design hinders the establishment of causal relationships, and the absence of data on asthma severity, individual-level income, and household-level environmental exposures may introduce unmeasured confounders. Despite adjusting for various covariates, there may still be residual confounding, and the study's timeframe (January 2018 to December 2020) may not capture long-term trends. These limitations should be considered when interpreting the results.

## Conclusion

In conclusion, the present study highlights the significant association between neighborhood socioeconomic status, measured by the Distressed Communities Index, and asthma-related ED visits in Maryland. Residents of economically distressed neighborhoods face a heightened risk of ED visits due to asthma, reflecting the complex interplay of healthcare access, environmental factors, and social determinants of health. These findings emphasize the need for targeted interventions to improve healthcar e access, reduce environmental pollution, and address health disparities in disadvantaged communities. By addressing these multifaceted challenges, we can work toward reducing the burden of asthma-related ED visits and improving the overall well-being of individuals living in vulnerable neighborhoods. Future research should delve deeper into the causal pathways linking neighborhood disadvantage and asthma outcomes, paving the way for more effective strategies to mitigate disparities and enhance asthma management.

## Data Availability

The original contributions presented in the study are included in the article/**Supplementary Material**. Requests to access these datasets should be directed to OA, austineakinyemi@gmail.com.
