# Supplementary material for: The relationship between neighborhood economic deprivation and asthma-associated emergency department visits in Maryland
Source: Front Allergy. 2024 Jun 5;5:1381184. doi: 10.3389/falgy.2024.1381184 (PMC11188351; doi:10.3389/falgy.2024.1381184)
Supplement: Supplementary file 1 [file Datasheet1.docx]

Supplementary 1: Asthma ICD-10 Diagnosis Codes

| J45.4 | J45.9 |
| --- | --- |
| J45.40 | J45.90 |
| J45.41 | J45.901 |
| J45.42 | J45.902 |
| J45.5 | J45.909 |
| J45.50 | J45.99 |
| J45.51 | J45.991 |
| J45.52 | J45.998 |

Supplementary 2. The 7 Metrics of the Distressed Communities Index

| 1 | The share of the population age 25 and older who lack a high school diploma or its equivalent |
| --- | --- |
| 2 | The share of individuals living below the federal poverty line |
| 3 | The share of the population aged 25 to 54 not working (i.e., either unemployed or not in the labor force) |
| 4 | The share of housing units that are vacant, adjusted for recreational, seasonal, or occasional use of vaccines |
| 5 | Median household income as a percent of the metro area or state median household income |
| 6 | The change from 2016 to 2020- in the number of employees working in the geography |
| 7 | The change from 2016 to 2020 in the number of establishments located in the geography |

Supplementary 3. Five Quintiles of the DCI and Respective Scores

| DCI Score | Quintile Name |
| --- | --- |
| 0-20 | Prosperous |
| 21-40 | Comfortable |
| 41-60 | Mid-Tier |
| 61-80 | At-Risk |
| 81-100 | Distressed |
